# Critical Breaths in Transit: A Review of Non-invasive Ventilation (NIV) for Neonatal and Pediatric Patients During Transportation

**DOI:** 10.3389/fped.2021.667404

**Published:** 2021-05-13

**Authors:** Nellie Ide, Grace Allen, Henry Charles Ashworth, Sara Dada

**Affiliations:** ^1^Department of Molecular and Cellular Biology, Harvard University, Cambridge, MA, United States; ^2^Department of Human Evolutionary Biology, Harvard University, Cambridge, MA, United States; ^3^Harvard Medical School, Boston, MA, United States; ^4^UCD Centre for Interdisciplinary Research, Education and Innovation in Health Systems, School of Nursing, Midwifery and Health Systems, University College Dublin, Dublin, Ireland

**Keywords:** non-invasive ventilation, respiratory intervention, emergency transportation, oxygen therapy, pediatric, neonatal, low resource

## Abstract

Respiratory illnesses are a leading cause of death for children worldwide, with the majority of these cases occurring from preterm birth complications or acute respiratory infections. Appropriate respiratory intervention must be provided quickly to lower the chances of death or permanent harm. As a result, respiratory support given in prehospital and interfacility transport can substantially improve health outcomes for these patients, particularly in areas where transportation time to appropriate facilities is lengthy. Existing literature supports the use of non-invasive ventilation (NIV), such as nasal or bilevel continuous positive airway pressure, as a safe form of respiratory support for children under 18 years old in certain transportation settings. This mini review summarizes the literature on pediatric NIV in transport and highlights significant gaps that future researchers should address. In particular, we identify the need to: solidify clinical guidelines for the selection of eligible pediatric patients for transport on NIV; explore the range of factors influencing successful NIV implementation during transportation; and apply appropriate best practices in low and middle income countries.

## Introduction

The response to the COVID-19 pandemic has increased efforts to expand oxygen availability worldwide. This wider availability has positive implications for pediatric clinical care beyond the pandemic (1). While children typically experience only minor symptoms from COVID-19, ~700,000 children globally under the age of five die each year due to lower respiratory infections such as bacterial and viral pneumonias (2, 3). Additionally, over 10% of newborns globally are born prematurely (4). Many of these newborns require respiratory support, as just minutes without adequate oxygen or ventilatory support can lead to permanent brain and lung injuries or death (5, 6). Improved oxygen availability alongside increased accessibility to treatments such as non-invasive ventilation (NIV) will be pivotal to decrease childhood mortality and morbidity from respiratory causes. Different forms of NIV, such as continuous positive airway pressure (CPAP), bilevel positive airway pressure (BiPAP), and nasal high flow therapy (nHFT), have been shown to safely and effectively provide respiratory support for children in respiratory distress in health facilities (7). When compared to invasive ventilation, NIV is simpler to apply, more cost-effective, and has less risks associated with it (8, 9).

In many regions of the world and particularly in low and middle income countries (LMICs), the transport time to a facility equipped to care for premature newborns or infants in respiratory distress may be hours long (10, 11). However, data on the use of NIV for children in transportation settings are minimal, and even more limited in LMIC contexts. As COVID-19 exacerbates existing healthcare inequities and leads to more children falling ill and mothers giving birth at home, it is more crucial than ever to increase research and attention on the use of NIV for children during prehospital and interfacility transport (12–14).

A systematic review published in 2018 synthesized eight studies on the use of NIV during pediatric critical care transport (15). The authors found that minor adverse events occurred in 1-4% of transfers, leading them to suggest that NIV in transport is safe in the settings studied (15). This review synthesizes additional literature on NIV during transportation and describes not only the safety considerations, but also the importance of standardized patient selection protocols and other factors that influence successful implementation. By analyzing new research and highlighting the current gaps in knowledge, this review provides actionable research questions for further investigation.

## Methods

**Figure 1:**
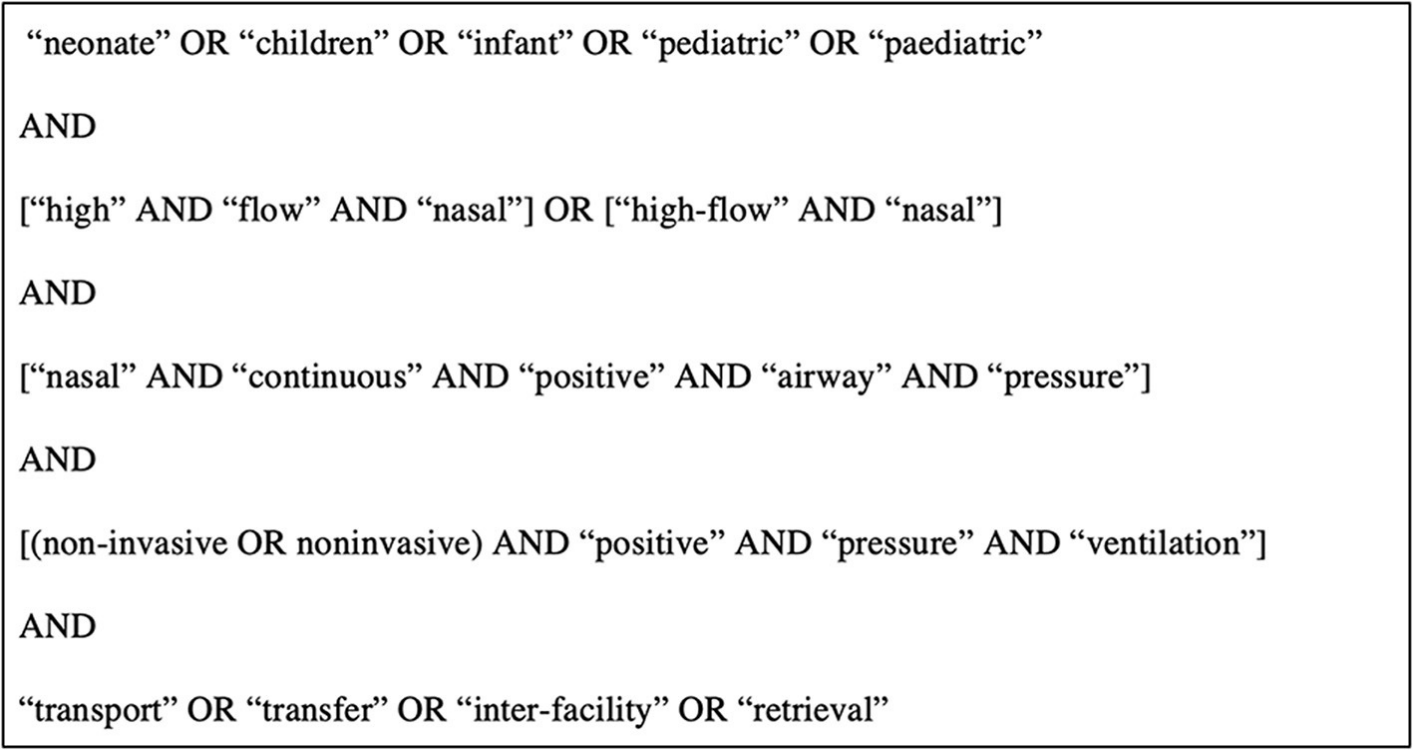
Search query.

The search query in Figure 1 was informed by the 2018 systematic review and used across the same databases (PubMed, EMBASE, Cochrane Central Register for Controlled Trials, African Index Medicus, and Web of Science) from the date of their search (March 15, 2017) through February 1st, 2021 (15). Additionally, Harvard Hollis library and Google Scholar were used to forward search any additional relevant references. Articles included in this study were limited to those with full text availability in English, and discussed the use of NIV in prehospital and transportation settings for children under 18 years old. Articles were excluded if they were not original research or if they were unrelated to NIV in a prehospital or transportation setting.

## Results

This mini review includes 15 papers that are cataloged in Table 1. Eight of these studies were included in Cheema et al.'s systematic review, five are additional observational studies, one is an epidemiological study of NIV use, and one is a qualitative survey. While the earliest article was published in 2005, the past decade has seen a steady increase in studies published on the topic.

**Table 1:** Summary table of included studies.

**References**	**Country**	**Type of NIV**	**Transport Setting**	Age of Patients^*^^*^as reported by study	**Number of participants**
Abraham et al. (16)	Australia	nHFT	Ground Air	Median (IQR): 28 days (13–51)	118
Baird et al. (17)	United States	BiPAP CPAP	Ground	Mean (SD): 6 yr (5) Mean (SD): 0.8 yr (0.7)	25
Bomont and Cheema (18)	United Kingdom	nCPAP^*^	Ground	Mean (SD): 28.3 d (24.3)	100
Fleming et al. (9)	Australia	CPAP	Ground	Mean (SD): 54 d (39)	51
Hansen et al. (19)	United States	BVM^†^	Ground	N/A	N/A
Holbird et al. (20)	Canada	HFNC^‡^ CPAP BiPAP	Ground	Mean (SD): 2.3 yr (3.6)	118
Jani et al. (21)	Australia	nCPAP	Ground	Preterm neonates—Median GA (range): 30 wk (27–31)	44
Millan et al. (22)	Spain	CPAP	Ground	Median (IQR): 3.4 mo (1.2–17)	108
Muniyappa et al. (23)	United States	nHFT	Ground Air	Median: <48 h	195
Murray and Stewart (24)	United Kingdom	nCPAP	Ground Air	Median (range): 1 d (0–175)	207
Resnick and Sokol (25)	Australia	CPAP	Ground	Median (range): 8 h (1–48)	166
Sheffield and Sheffield (26)	Canada	nCPAP	Air	Mean: 21 d	5
Schlapbach et al. (27)	Australia	HFNC	Ground	Mean (range): 6.5 mo (0–24)	160
Trevisanuto et al. (28)	Italy	HFNC CPAP BiPAP	Ground	Range: At birth to >30 days	3,337
Zein et al. (29)	Canada	nCPAP	Ground	Preterm neonates—Median GA (IQR): 28 wk (26–29)	99

* *Nasal CPAP*,

† *Bag valve mask manual resuscitation*,

‡ *High flow nasal cannula*.

### Safety

Cheema et al.'s systematic review of eight studies suggested that NIV is safe to use in transport for children under age 18 (15). Three of 858 patients (0.4%) required escalation of the mode of ventilation during transport (15). Five additional studies in our review demonstrated the safety of NIV in lengthy ground and air transport, in rural settings, and for preterm neonates outside the hospital setting (16, 20, 23, 26, 29). In one study, none of 118 pediatric patients on NIV during transport required intubation, despite a mean transport time of 163 min (20). Two studies conducted in remote settings suggested that nCPAP and nHFT are safe in air transport (23, 26). Another study reported no adverse events in the interfacility transfer of 99 preterm neonates on nCPAP and concluded that this practice was both safe and cost-effective (29).

Use of NIV in transportation settings decreases the need for invasive ventilation and consequently avoids the risks associated with this practice (8, 9, 16, 27). Invasive ventilation requires monitoring and sedation and increases the risk of potential lung damage from barotrauma (9). Additionally, patients with comparable disease severity have longer hospital stays when managed with invasive ventilation compared with NIV (9). In contrast, NIV is simpler to apply and can be easily discontinued when necessary (8). A study conducted in Australia on interhospital transfer of children under 2 years old found that prior to introduction of high flow nasal cannula (HFNC) in transport, 49% of the children were transported on invasive ventilation (27). After introduction of HFNC, this percentage decreased to 33% (27). Additionally, none of the patients transported on HFNC required intubation during transport (27). These results were supported by other studies, which described that although many patients did not require invasive ventilation, most would have been intubated during transport due to a lack of other treatment options (9, 25, 26). With the increasing use of NIV in transport, more of these children can avoid invasive ventilation (28).

While these studies suggest that NIV is safe to use during transport for children in their respective settings, none were conducted in LMICs. Further research should investigate the safety of NIV in LMICs. Once safety has been demonstrated in low-resource settings, a shift in focus toward implementation research may help make widespread use more feasible.

### Patient Selection

Many studies included in this review described the challenge of determining the best method of ventilatory support for a patient in transport and emphasized the need for evidence-based clinical guidelines (9, 18–20, 22, 23, 25). This decision requires weighing the risks of providing inadequate ventilation using NIV vs. the risks of complications from invasive ventilation (22). Beyond clear contraindications for NIV such as a pneumothorax, cardiorespiratory arrest, and a complete upper airway obstruction, there are few agreed upon indicators for the selection of patients for NIV in transport (8, 17, 22). This review suggests three parameters that could be included in a protocol for selection of NIV-eligible patients: (1) a limited stabilization period before transport, (2) a threshold for the required fraction of inspired oxygen (FiO_2_), and (3) a threshold for the blood hemoglobin oxygen saturation (SpO_2_)/FiO_2_ ratio (21–25).

The first parameter is the stabilization period, the length of time during which a patient adjusts to a new treatment and is monitored for treatment effectiveness before beginning interfacility transport (9, 21, 22). One study found that patients transported on nHFT who required an increase in flow rate (≥2 L/min above the starting flow rate) and/or an increase in the FiO_2_ (≥0.20 above the starting FiO_2_) during transport had statistically significant longer stabilization periods (56 ± 25 min) than the patients who did not (39 ± 18 min) (23). While requiring increases in nHFT parameters does not signify a failure of nHFT in transport, it may indicate that a patient's condition is deteriorating or not adequately treated by NIV (23). This preliminary difference in the stabilization period suggests that with additional evidence, the length of a stabilization period can be used to identify patients who may need a different method of ventilation. Another study required significant improvement in a patient's severity score^1^ within 30 min of treatment, otherwise the method of ventilation was changed before beginning transport (22). Using this requirement, the mean stabilization time for patients transported on NIV was 48 min (22). Jani et al. used a more flexible method and observed patients for “~30 min or until stabilization” to determine whether it was necessary to escalate the method of ventilation (21). It appears that a limited stabilization period may help identify if escalation of ventilation is required prior to transport, but at present there is no consensus on how long this stabilization time should be.

Multiple studies suggested using the required FiO_2_ to determine the best method of ventilation for children (21, 23–25). A study of 166 newborns transported on nCPAP compared key measurements of disease severity between patients who required intubation within 24 h of transport and those who did not (25). FiO_2_ prior to transport was the only measurement with a statistically significant difference between these two groups (25). Patients who were intubated within 24 h of transport required higher FiO_2_ prior to transport (median = 0.55) and their oxygen requirement decreased only minimally after CPAP was administered (median = 0.02) (25). In contrast, patients transported on CPAP without requiring intubation required a lower FiO_2_ (median = 0.45) and had a larger decrease in their oxygen requirement (median = 0.13) (25). Similarly, Murray and Stewart found that patients who were intubated within 24 h of transport had a mean initial FiO_2_ of 0.5, while patients who were not intubated had a mean initial FiO_2_ of 0.37 (24). Thus, Murray and Stewart recommended intubating patients if they require an FiO_2_ higher than 0.45 prior to transport and if this FiO_2_ requirement is not reduced within 20 min of CPAP initiation (24). However, there was no consensus across papers on what the FiO_2_ threshold should be.

The third parameter, the SpO_2_/FiO_2_ (S/F) ratio, compares the SpO_2_ and FiO_2_ measurements to determine the severity of respiratory distress (30). Muniyappa et al. found that the S/F ratio was significantly lower for patients who failed NIV in transport compared to those who did not (23). In another study, in cases where acute respiratory distress syndrome was suspected, an S/F ratio <150 after 30 min of NIV treatment was used as a contraindication for the continuation of NIV in transport (22). A study on the use of HFNC for children in the hospital setting used a different S/F ratio threshold and recommended escalating the mode of ventilation if an S/F ratio was <200 after 60 min of HFNC (30). Further research is needed to determine an optimal S/F ratio threshold for the use of different NIV methods on children in transportation settings.

These three parameters (stabilization period, FiO_2_ level, and S/F ratio) could provide a starting point to guide patient selection for NIV during transportation. While decisions may differ based on diagnosis, patient ages, transport distances, and available resources, development of evidence-based clinical pathways is an important area for further work.

### Implementation

In order to effectively use NIV during pediatric transport, it is essential to consider factors that influence implementation. For example, NIV devices integrated into medical transport settings must be well-suited to the unique challenges presented by ground or air transportation. It is particularly important to consider durability, weight, size, and compatibility with various power sources and other respiratory care equipment (8).

Multiple studies in this review emphasized the importance of training and experience to successfully integrate NIV into pediatric transport (17–20). A survey of prehospital providers found that they had low confidence with pediatric respiratory emergencies, particularly regarding patient assessment and management (19). Anxiety with pediatric respiratory emergency care increases the likelihood of medical errors, especially in critically ill cases (19). Given the high level of skill and experience required for use of NIV in transport, nine out of the 12 observational studies in this review described use of a specialized transport team (9, 17, 18, 20–24, 27). These specialized transfer units usually consist of a transport nurse and physician and/or respiratory therapist with significant training and experience in the field (18, 21, 23). In one study, paramedics in an interhospital transport service underwent 200 h of training on adult and pediatric transport medicine, which included 5 h of training on NIV and 2 h on advanced pediatric airway skills (17). These rigorous programs increased team members' exposure to the practice of pediatric NIV which gave the teams more confidence in deciding which patients may benefit from NIV, applying NIV to these patients, and managing most complications (19, 23). While specialized transport teams may not be available in low-resource health systems, additional training on pediatric NIV may facilitate the use of NIV during transport in these settings.

### NIV During Transportation in LMICs

While we did not find any research in LMICs on the use of NIV in transportation settings, previous work on the effectiveness of bCPAP in LMIC hospitals called for further investigation into transportation and highlighted it as a key link to improve respiratory care (31, 32); (Dada et al. under review). In LMICs, additional factors such as long transport times, limited availability of appropriate training, limited resources in health facilities, and low population density must be considered in the implementation of NIV (23, 26). While our literature search did not identify any studies conducted in LMICs, two studies conducted in rural settings in Canada and Utah cited barriers similar to what may be expected in LMICs (23, 26). Despite lengthy travel times, all treated infants arrived in stable condition and no adverse events were recorded in these two studies (23, 26). Although in both studies providers had access to air transport and specialized transfer teams, the results suggest that implementation of NIV in transport in LMICs may be feasible. Additionally, Zein et al. showed that the use of nCPAP to transfer patients from a level three hospital to a level two neonatal intensive care unit (NICU) saved 2.65 million Canadian dollars and freed up at least 848 days of NICU beds (29). This suggests that using NIV in transport for children may have financial benefits that could help maximize the use of limited resources in LMICs. Research in low resource settings is needed to understand barriers and facilitators to quality NIV treatment during transportation.

## Discussion

The observational studies included in this review primarily focused on the safety of NIV for children in transportation settings (15). These studies endorsed the use of NIV to provide safe and adequate respiratory support and to avoid additional risks associated with invasive ventilation (9, 17, 18, 20–27, 29). Future research should determine the requirements for healthcare systems to safely introduce NIV into transportation systems. Once this is established, other areas can be investigated, such as comparing the efficacy of different modes of NIV in transportation. To aid in this effort, a standard protocol must be developed to identify patients eligible for successful NIV in transport. While there is some consensus on the parameters to include in a protocol, the included studies primarily focus on term and preterm infants, and it is unclear if these parameters are applicable to older children. Thus, further studies are needed to better understand optimal stabilization periods and FiO_2_ and S/F ratio thresholds, and to adapt these findings to different age groups, diagnoses, and NIV methods (21–25). Lastly, it is important to note that assessing these metrics requires technology, such as pulse oximeters, that is not always available in low-resource settings or in transportation vehicles. The process for patient selection may need to include decision trees based on limited available resources.

The current literature does not adequately address the preconditions necessary to integrate NIV into the transportation segment of a health system. For example, the need for NIV devices compatible with transportation vehicles appears clear, but the exact requirements for successful introduction of NIV into new settings are not well-understood (8). Similarly, many studies emphasized the necessity of a well-trained team, but very few studies reported details on the training processes (17–20). More research is needed to determine how medical transport education can be developed to support successful NIV.

Finally, the lack of NIV studies from LMICs impedes understanding of the extent to which NIV is being used for children in transportation in these settings, if at all, and the degree to which NIV, if being used, is improving pediatric patient outcomes. With 60% of LMIC populations residing more than eight kilometers from healthcare facilities, long transport times should be an additional consideration in the implementation of NIV in LMICs (33). To date, studies conducted in rural settings with mean transport distances up to 236 kilometers suggest that this barrier can be overcome (20, 23, 26). However, in many LMICs <1% of people have access to emergency medical transportation services, resulting in patients relying on their own means of transportation (33). When combined with limited access to oxygen, it must be acknowledged that in some areas it may not be possible to use NIV in transportation. It is essential for new studies to investigate what is necessary for safe and effective implementation of NIV in transportation in LMICs.

Overall, data on NIV for children in transportation are limited by the ethical complications of working with these high-risk populations. For example, researchers are unable to conduct randomized controlled trials to determine the effectiveness of NIV in various settings (15). As previous studies have stated, the existing data are challenging to synthesize due to the variety in methods, metrics, and patient populations (15, 23). We did not conduct a systematic review or critical appraisals of the studies in this review. We also note that limited sample sizes and practice biases could affect the quality of the studies' results.

## Conclusion

NIV for newborns and children appears safe to use in high resource transportation settings and can even improve patient outcomes. More research is needed to develop protocols for optimizing patient selection and to inform large-scale implementation strategies. Implementation of NIV in pediatric transport depends on resources and conditions within healthcare systems. In high-resource settings, implementation efforts may focus on refining current practices, such as utilization of specialized transport teams. In LMICs there are additional challenges to overcome, but little is currently known about the barriers and facilitators to optimal use of NIV in transport in these settings. Such information would enable efforts to integrate NIV for children into more prehospital and interfacility transport settings around the world.

## Author Contributions

NI and GA conducted the literature search, analyzed the data, and drafted the manuscript. HA and SD conceived the idea for this review, advised on the design, and provided edits. All authors contributed to the article and approved the manuscript prior to submission.

## Conflict of Interest

The authors declare that the research was conducted in the absence of any commercial or financial relationships that could be construed as a potential conflict of interest.

## Footnotes

^1^Millan et al. used the Wood-Downes severity score for patients with asthma, and the BROSJOD score for patients with bronchiolitis.
